# PAX7 target gene repression is a superior FSHD biomarker than DUX4 target gene activation, associating with pathological severity and identifying FSHD at the single-cell level

**DOI:** 10.1093/hmg/ddz043

**Published:** 2019-03-13

**Authors:** Christopher R S Banerji, Peter S Zammit

**Affiliations:** 1 King’s College London, Randall Centre for Cell and Molecular Biophysics, New Hunt’s House, Guy’s Campus, London, UK; 2 Faculty of Medicine, Imperial College London, Level, Faculty Building, South Kensington Campus, London, UK

## Abstract

Facioscapulohumeral muscular dystrophy (FSHD) is a prevalent, incurable skeletal myopathy. The condition is linked to hypomethylation of the D4Z4 macrosatellite repeat at chromosome 4q35, leading to epigenetic derepression of the transcription factor DUX4; coupled with a permissive 4qA haplotype supplying a poly(A) signal. DUX4 may drive FSHD pathology via both induction of target genes and inhibition of the function of the myogenic master regulator PAX7. Biomarkers for FSHD have focused on DUX4 target gene expression. We have, however, reported that PAX7 target gene repression is a hallmark of FSHD skeletal muscle. Here we demonstrate that PAX7 target gene repression is an equivalent biomarker to DUX4 target gene expression when considering RNA-Sequencing data from magnetic resonance imaging-guided muscle biopsies. Moreover, PAX7 target gene repression correlates with active disease, independent to DUX4 target gene expression. PAX7 target genes are also repressed in RNA-Sequencing data from single cells, representing a significantly better biomarker of FSHD cells than DUX4 target gene expression. Importantly, PAX7 target gene repression is a significant biomarker in the majority of FSHD cells that are DUX4 target gene negative, and on which the DUX4 biomarker is indiscriminate. To facilitate the evaluation of validated biomarkers we provide a simple tool that outputs biomarker values from a normalized expression data matrix. In summary, PAX7 target gene repression in FSHD correlates with disease severity, independently of DUX4 target gene expression. At the single-cell level, PAX7 target gene repression can efficiently discriminate FSHD cells, even when no DUX4 target genes are detectable.

## Introduction

Facioscapulohumeral muscular dystrophy (FSHD) is a prevalent [12/100 000 (1)] inheritable skeletal myopathy. Clinically, FSHD typically presents as a skeletal muscle weakness and atrophy beginning in the facial muscles, before descending to the shoulder girdle and finally muscles of the lower limb; in a characteristic distribution (2). Unlike most other muscular dystrophies though, there is often a marked left–right asymmetry in the degree that muscles are affected. FSHD is also highly heterogeneous, where presentations can vary dramatically between first-degree relatives and even monozygotic twins (3,4). There is also a differential penetrance between males and females, with males generally presenting earlier in life (5). In addition to myopathy, FSHD associates with a retinal telangiectasia (6) and/or sensorineural hearing loss in a subset of patients (7).

FSHD is linked to hypomethylation of the D4Z4 macrosatellite repeat at chromosome 4q35, alongside a permissive 4qA haplotype. Hypomethylation is achieved either by truncation of the D4Z4 macrosatellite to between 1 and 10 D4Z4 repeats (FSHD1 - MIM 158900) (8) or via mutation in the chromatin remodelling gene *SMCHD1* (9) or in rare cases, by mutations in *DNMT3B* (FSHD2 - MIM 158901) (10). Each 3.3 kb D4Z4 repeat contains a retro-transposed open reading frame encoding a transcription factor termed double homeobox 4 (DUX4) (11,12). DUX4 is normally expressed at the four-cell human embryo phase, where it activates a cleavage-stage transcriptional program (13,14). In FSHD though, D4Z4 hypomethylation results in epigenetic derepression, allowing generation of DUX4 transcripts from the most distal D4Z4 unit. DUX4 RNA is then polyadenylated by the poly(A) signal in the flanking pLAM region of permissive 4qA haplotypes, allowing expression of DUX4 protein in FSHD patients (8,15). Thus, ectopic DUX4 expression likely underlies FSHD pathogenesis in both FSHD1 and FSHD2 (16).

Investigations into how DUX4 drives FSHD pathology have largely focused on the expression of DUX4 target genes, some of which have been demonstrated to be anti-myogenic (17–19) and pro-apoptotic (20–22). However, DUX4 is notoriously difficult to detect in FSHD, with expression reported to be as low as 1/1000–1/5000 myoblasts and 1/200 nuclei in differentiated myotubes, and in only 50% of FSHD muscle biopsies (23–26). An alternate hypothesis derives from the fact that the homeodomains of DUX4 demonstrate significant amino acid homology to those of the myogenic transcription factor PAX7 (20). Indeed, DUX4 homeodomains can be substituted with those of PAX7 without affecting certain DUX4 functions (27). Such homology explains competitive inhibition between DUX4 and PAX7 in activating their respective transcriptional target genes in human cells *in vitro* (28), while in murine myoblasts, PAX7 over-expression, for example, can rescue DUX4-mediated apoptosis (20).

Identification of biomarkers for FSHD is of importance for elucidation of molecular mechanisms, development of therapies and as a surrogate, quantitative outcome measure for clinical trials. Several biomarkers have been proposed, focused on DUX4 target gene expression and genes found to be differentially expressed in FSHD muscle biopsies (29,30). We recently performed a head-to-head comparison of published transcriptomic biomarkers alongside a novel biomarker based on PAX7 target gene repression in a meta-analysis across six independent data sets [five microarray-based and one limited RNA-Sequencing (RNA-Seq)] profiling FSHD and control skeletal muscle (28). Only the PAX7 target gene repression biomarker could discriminate FSHD from control muscle biopsies on every data set considered. In contrast, DUX4 target gene expression signatures were only discriminatory on a microarray study of magnetic resonance imaging (MRI)-guided muscle biopsies and the single limited RNA-Seq data set (28). We confirmed that PAX7 target genes were suppressed in FSHD immortalized myoblasts, and DUX4 target genes were over-expressed. However, without single-cell resolution, we were unable to determine how the two molecular mechanisms interact.

Two new RNA-Seq transcriptomic studies have recently been published on FSHD (31,32). The first study profiled 34 FSHD muscle biopsies obtained under MRI guidance and 9 controls, alongside detailed histopathological characterization of the profiled muscle, allowing gene expression to be correlated to pathological severity (31). The second study performed single-cell RNA-Seq on primary myocyte cultures from two FSHD1 patients, two FSHD2 patients and two controls (32). These studies demonstrated that DUX4 was a significant biomarker of FSHD status and associated with disease severity, but only used expression of derivatives of a single patent-pending DUX4 target gene biomarker. However, neither study examined the status of PAX7 target gene repression. Thus, it is unclear whether PAX7 target gene repression correlates with histopathological markers of disease severity, independently to DUX4 target genes, which could identify PAX7 target genes as an independent pathomechanism for therapeutic targeting. Moreover, the single-cell study found only 0.4% (23/5133) of FSHD cells expressing five or more DUX4 target genes (32), raising the question as to whether PAX7 target gene repression may more reliably discriminate DUX4 target gene-negative FSHD cells from controls, and hence represent a more efficient biomarker.

Our aim here was to examine how our PAX7 biomarker correlates with FSHD disease severity and its ability to discriminate FSHD at the single-cell level. We found that PAX7 target gene repression was as strong an FSHD biomarker in the RNA-Seq of MRI-guided skeletal muscle biopsies as both proprietary and our two non-proprietary DUX4 target gene expression biomarkers. Importantly, PAX7 target gene repression was correlated with histopathological measures of FSHD disease activity in a manner independent to DUX4 target gene expression. Muscle biopsies with high PAX7 target gene repression and high DUX4 target gene expression had more than double the disease activity than samples with low PAX7 target gene repression and high DUX4 target expression. At the single-cell level, PAX7 target gene repression was also a significantly more reliable biomarker of FSHD cells than DUX4 target gene expression. Of FSHD myocytes, 19.7% express DUX4 target genes, and we again find that PAX7 target gene repression is a significant biomarker. Crucially, however, of the 80.3% of FSHD myocytes that expressed no DUX4 target genes, so making them indistinguishable from control cells, we found that PAX7 target gene repression remained a significant biomarker of FSHD status. To facilitate consideration of PAX7 target gene repression in the analysis of FSHD transcriptomic data, we provide a simple pipeline for extraction of PAX7 target gene repression, as well as three validated DUX4 target gene expression biomarkers, from normalized gene level expression data (Supplementary Material, File S1).

Together, our results show that PAX7 target gene repression increases with FSHD disease severity, consistent with a role for these genes in driving pathology. At the single-cell level, PAX7 target gene repression can discriminate FSHD cells from controls, even when these cells are DUX4 target gene negative, suggesting that this biomarker may be of greater use than DUX4 target gene expression. This may be especially useful when samples are rare and quantity is low, as in the case of painful muscle biopsies for patients with muscular dystrophy.

## Results

### PAX7 target gene repression is an equivalent biomarker to DUX4 target gene expression on MRI-guided FSHD muscle biopsies

Normalized gene counts describing RNA-Seq performed on 34 FSHD skeletal muscle biopsies of the lower limb selected by MRI, alongside 9 matched controls, were obtained from the GEO database (33), accession GSE115650 (31).

**Figure 1:**
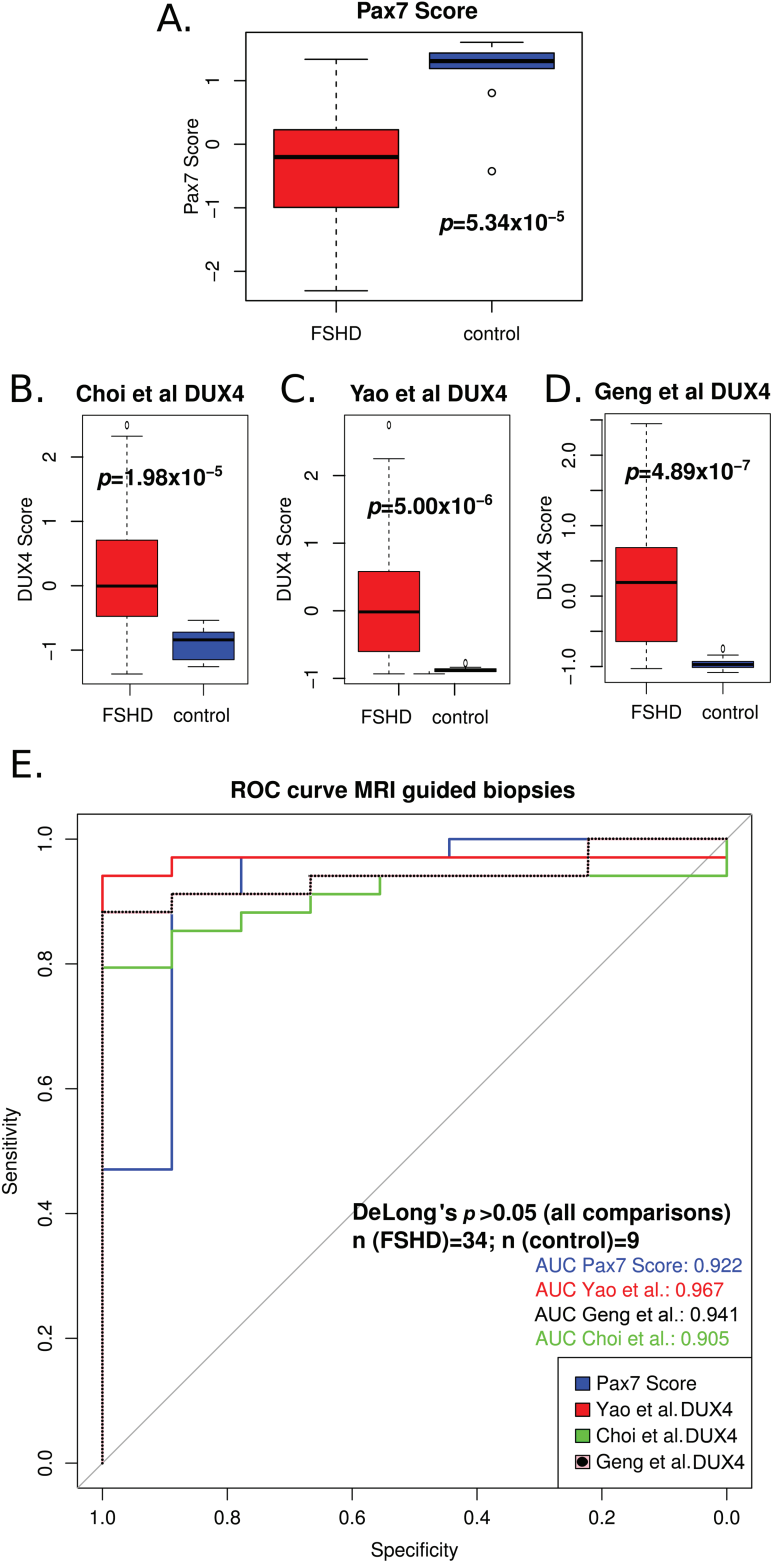
PAX7 target gene repression is an equivalent biomarker to DUX4 target gene expression on MRI-guided FSHD muscle biopsies. (**A**) A box plot demonstrates that the PAX7 target gene signature derived from 311 up-regulated target genes and 290 down-regulated target genes in mouse validates as a biomarker on the MRI-guided RNA-Seq FSHD skeletal muscle biopsy data set published by Wang *et al*. (2019) (31). The box represents the interquartile range (IQR), with the median indicated by a line. Whiskers denote min[1.5^*^IQR, max(observed value)]. ‘o’ represents data points >1.5 IQR from the median. *n* = 34 FSHD and *n* = 9 control muscle biopsies. The two-tailed Wilcoxon U-test *P-*value is given. (**B**–**D**) Box plots demonstrate that the DUX4 target gene expression biomarkers of Yao *et al*. (2014) (30) and two further DUX4 signatures that we derived previously (28) from studies by Geng *et al*. (2012) (24) and Choi *et al*. (2016) (34) validate as biomarkers on the MRI-guided RNA-Seq FSHD muscle biopsy data set published by Wang *et al*. (2019) (31). Boxes represent the IQR, with the median indicated by a line. Whiskers denote min[1.5^*^IQR, max(observed value)]. ‘o’ represents data points >1.5 IQR from the median. *n* = 34 FSHD and *n* = 9 control muscle biopsies. (**E**) A ROC curve compares the discriminatory power of our PAX7 biomarker with the DUX4 target gene signatures from Yao *et al*. (2014) (30), Geng *et al*. (2012) (24) and Choi *et al*. (2016) (34), across the MRI-guided RNA-Seq FSHD muscle biopsy data set published by Wang *et al*. (2019) (31) (*n* = 34 FSHD and *n* = 9 control muscle biopsies). DeLong’s test *P-*value demonstrates that the PAX7 and each DUX4 biomarker are equally excellent discriminators of FSHD status.

We first computed our PAX7 target gene repression biomarker derived from 311 up-regulated PAX7 target genes and 290 down-regulated PAX7 target genes (28); PAX7 target genes were significantly repressed in FSHD samples compared to controls (Wilcoxon *P* = 5.34 × 10^−5^; Fig. 1A). We next computed DUX4 target gene expression using the full Yao *et al*. (2014) (30) 114 DUX4 target gene expression biomarker (patent application number: WO2015143062A1) and two further DUX4 target gene expression biomarkers that we derived previously (28): comprising 165 DUX4 target genes from Geng *et al*. (2012) (24) and 212 DUX4 target genes from Choi *et al*. (2016) (34). All three DUX4 target gene expression biomarkers showed elevated expression on the FSHD samples compared to controls (Wilcoxon *P* < 2 × 10^−5^; Fig. 1B–D). DUX4 target gene expression was previously assessed on this data set by Wang *et al*. (2019) (31) using a subset of either 4 (*TRIM43*, *LEUTX*, *PRAMEF2* and *KHDC1L*) or 54 of the 114 DUX4-induced genes identified by Yao *et al*. (2014) (30), rather than the full biomarker, limiting comparison with prior studies. Wang *et al*. (2019) (31) also described a subset of 10 FSHD samples that expressed similar levels of their 4 DUX4 target genes to controls, and were thus indistinguishable. When we considered these 10 samples, we found that PAX7 target gene repression (*P* = 0.0015) was able to discriminate these FSHD samples from controls. Interestingly, all 3 full DUX4 target gene biomarkers (*P* = 0.0030) were also able to distinguish these 10 samples, emphasizing the value of also using the full validated DUX4 biomarkers.

We next performed quantification and comparison of the four biomarkers using receiver operating characteristic (ROC) curve analysis. Essentially, a ROC curve depicts the performance of a binary classifier at different threshold values. In this case, the biomarkers are binary classifiers of FSHD status. The true positive rate (sensitivity) of the biomarker classifier, is plotted against the false positive rate (1-specificity) at different biomarker threshold values to generate the ROC curve. The area under the curve (AUC) represents the probability that the biomarker being plotted will on average discriminate an FSHD sample from a control. An uninformative classifier will have an AUC = 0.5, equivalent of classifying samples randomly (e.g. by flipping an unbiased coin), while an AUC > 0.5 implies an informative classifier. The higher the AUC above 0.5, the better the biomarker is at distinguishing FSHD from healthy, and AUC = 1 implies perfect separation of biomarker value distributions on FSHD and control samples. The PAX7 target gene repression and three DUX4 biomarkers performed well, each having an AUC > 0.9 (Fig. 1E). To directly compare the relative effectiveness of the four ROC curves constructed from the four biomarkers on the same muscle biopsies, we used a non-parametric DeLong’s test (35) to analyse and compare the AUC for each biomarker. PAX7 target gene repression was indistinguishable as a biomarker from the three based on DUX4 target gene expression (DeLong’s test *P* > 0.05), in line with our previous findings on RNA-Seq data from FSHD biopsies (28).

### PAX7 target gene repression correlates with histopathological measures of disease activity independently of DUX4 target gene expression

**Figure 2:**
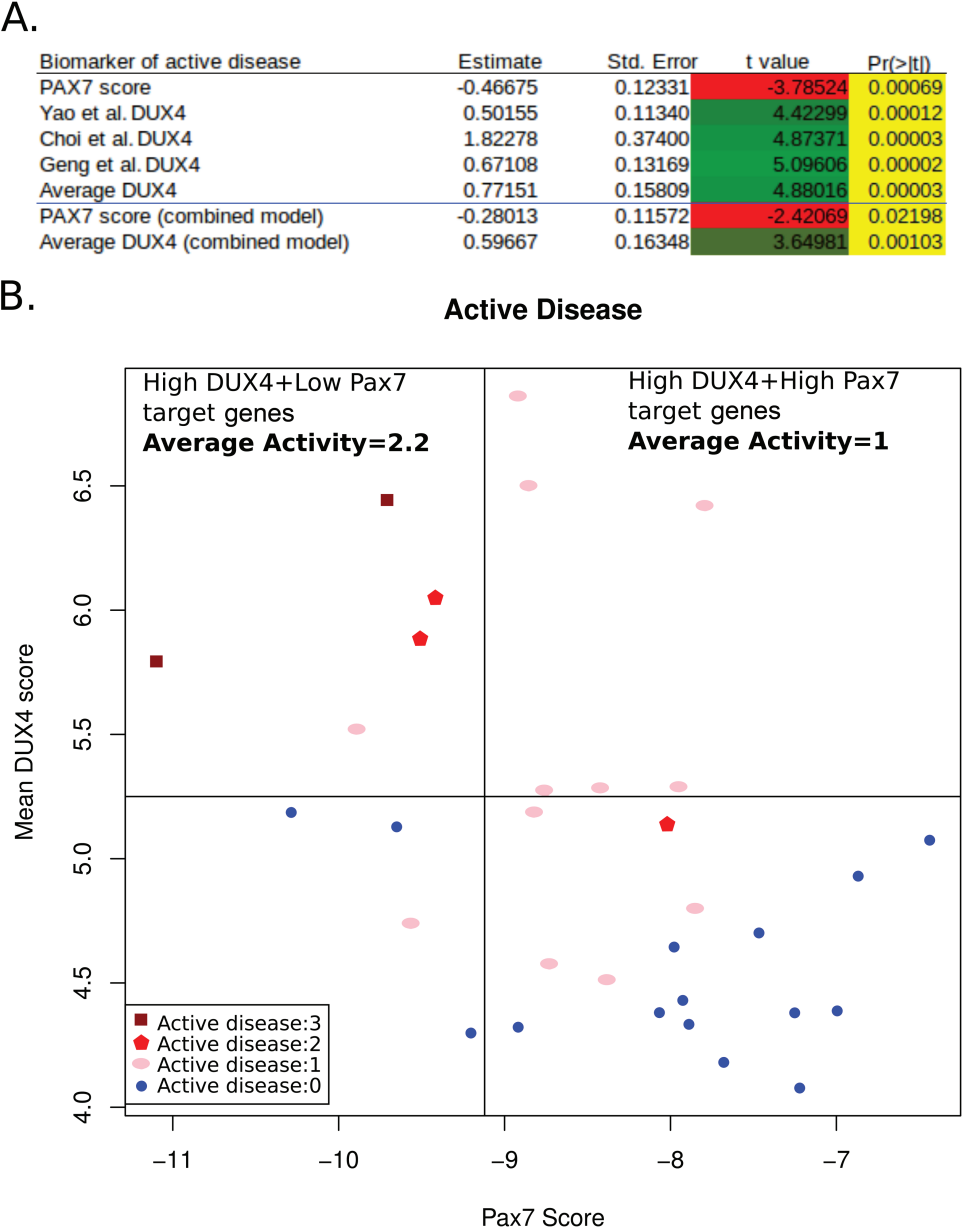
PAX7 target gene repression correlates with histopathological measures of disease activity independently of DUX4 target gene expression. (**A**) Table displaying summary statistics for linear models fitting histopathological disease activity to the various FSHD biomarkers. The first five rows of the table show statistics for the PAX7 target gene repression biomarker, and each of the three DUX4 target gene expression biomarkers, as well as the average of the three DUX4 target gene expression biomarkers. All are significantly associated with histopathological disease activity. The last two rows show the statistics for a multivariate linear model fitting disease activity to both average DUX4 target gene expression and PAX7 target gene repression; we see that both biomarkers are independently associated with active disease (Pr(>|t|) value). (**B**) A scatter plot of mean DUX4 target gene expression biomarker against the PAX7 target gene repression biomarker for the 32 FSHD patient MRI-guided muscle biopsies for which histopathological assessment of active disease was made. All patients with high mean DUX4 target gene expression above 5.25 show evidence of active disease; however, among these samples, those with high PAX7 target gene repression below −9.12 demonstrate 2.2 times higher activity levels compared to those with low PAX7 target gene repression.

Wang *et al*. (2019) (31) also combined gene expression analysis using RNA-Seq with histological and MRI-based read-outs of disease activity in the FSHD samples used for transcriptomics. In particular, the authors demonstrated that DUX4 target gene expression associated with active disease, as assessed by histology and short-TI inversion recovery (STIR) and T1 positivity on MRI (31). We assessed the association between PAX7 target gene repression, as well as the three DUX4 target gene expression biomarkers separately, on these measures of disease severity. DUX4 target gene expression was significantly associated with both histological (pathology score *P* < 5.0 × 10^−4^, inflammation *P* < 1.3 × 10^−5^, active disease *P* < 1.2 × 10^−4^) and imaging measures of disease severity (STIR+ *P* < 5.5 × 10^−4^, T1+ *P* < 2.2 × 10^−3^, fat fraction *P* < 1.3 × 10^−3^). PAX7 target gene repression showed significant association with the histological measures (pathology score *P* < 5.3 × 10^−3^, inflammation *P* < 9.6 × 10^−5^, active disease *P* < 6.9 × 10^−4^) and trended towards significance with the imaging measures (STIR+ *P* = 0.053, T1+ *P* = 0.085, fat fraction *P* = 0.11). Linear models fitting disease activity, as assessed by histopathology, to the various FSHD biomarkers are shown in Fig. 2A. However, when a multivariate model was considered, PAX7 target gene repression and DUX4 target gene expression were independently associated with histological assessment of active disease (PAX7 co-efficient *P* = 0.022, DUX4 co-efficient *P* = 0.001; Fig. 2A) and specifically inflammation (PAX7 co-efficient *P* = 0.0027, DUX4 co-efficient *P* = 0.00071). This implies that both PAX7 target gene repression and DUX4 target gene expression contribute to the level of active disease. A mean DUX4 target gene expression level of >5.25 was sufficient to ensure samples displayed active disease (Fig. 2B). When these samples were divided into two groups on the basis of PAX7 target gene repression, however, those with high PAX7 target gene repression below −9.12 (mean value of high DUX4 samples) had 2.2 times greater disease activity compared to those with lower PAX7 target gene repression (Fig. 2B). Thus, PAX7 target gene repression and DUX4 target gene expression may act synergistically to drive active disease in FSHD. Indeed, both biomarkers employed in tandem can stratify patients more accurately into those with highly active disease than a single biomarker alone can achieve.

**Figure 3:**
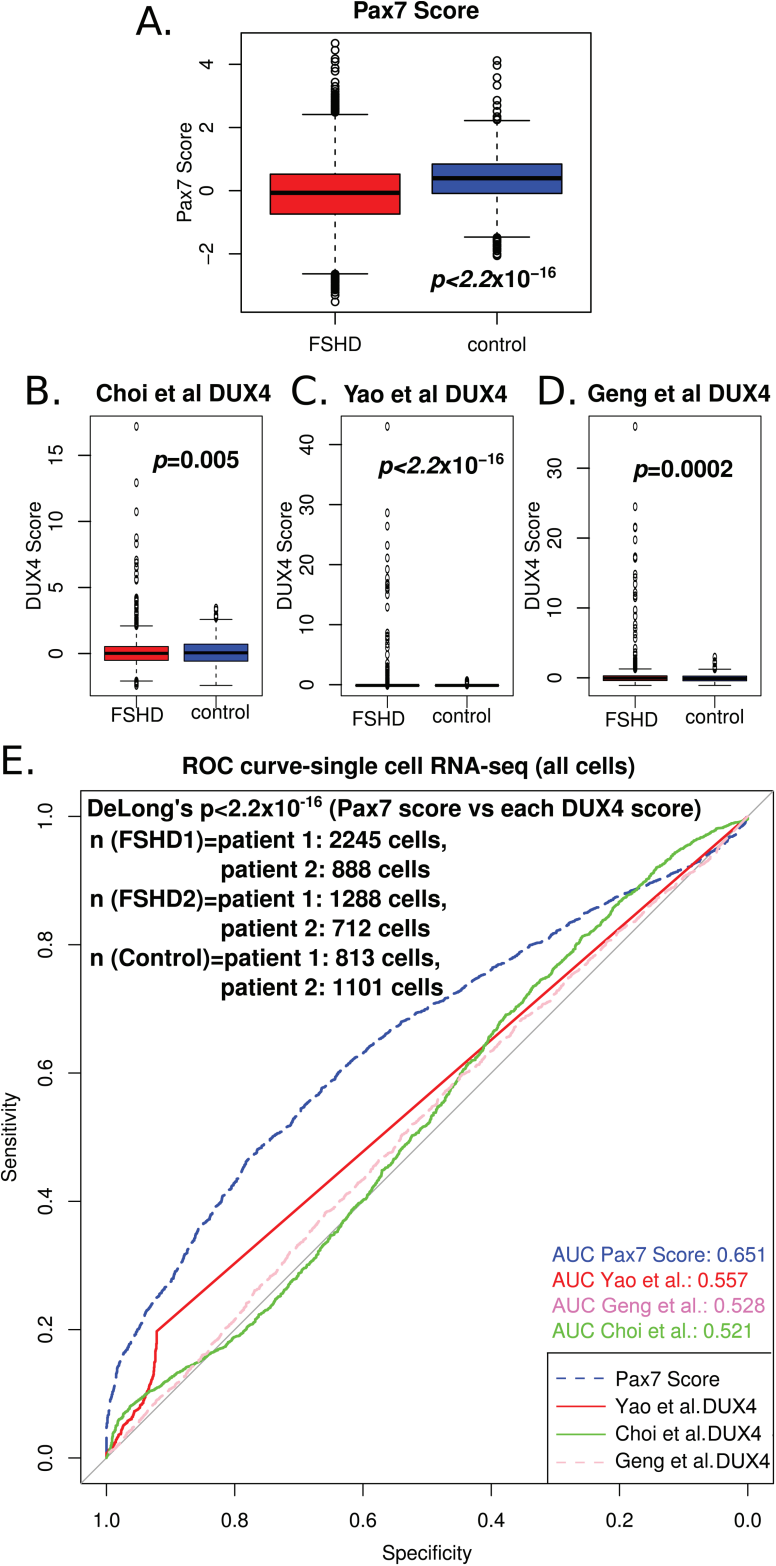
PAX7 target gene repression is a superior biomarker to DUX4 target gene expression in single-cell RNA-Seq of FSHD myocytes*.* (**A**) A box plot demonstrates that the PAX7 target gene signature derived from 311 up-regulated target genes and 290 down-regulated target genes in mouse validates as a biomarker in 7047 myocytes sequenced from two FSHD1, two FSHD2 and two control individuals, published by van den Heuvel *et al*. (2019) (32). The box represents the IQR, with the median indicated by a line. Whiskers denote min[1.5^*^IQR, max(observed value)]. ‘o’ represents data points >1.5 IQR from the median. The two-tailed Wilcoxon U-test *P-*value is given. (**B**–**D**) Box plots demonstrate that the 114 DUX4 target gene expression biomarker of Yao *et al*. (2014) (30), and two further DUX4 signatures that we derived previously (28) from studies by Geng *et al*. (2012) (24) and Choi *et al*. (2016) (34), each validate as biomarkers on the 7047 cells sequenced from two FSHD1, two FSHD2 and two control individuals, published by van den Heuvel *et al*. (2019) (32). Boxes represent the IQR, with the median indicated by a line. Whiskers denote min[1.5^*^IQR, max(observed value)]. ‘o’ represents data points >1.5 IQR from the median. (**E**) A ROC curve compares the discriminatory power of our PAX7 biomarker with the DUX4 target gene signatures of Yao *et al*. (2014) (30), Geng *et al*. (2012) (24) and Choi *et al*. (2016) (34), across the 7047 cells sequenced from two FSHD1, two FSHD2 and two control individuals, published by van den Heuvel *et al*. (2019) (32). DeLong’s test *P-*value (*P* < 2.2 × 10^−16^) demonstrates that the PAX7 biomarker (AUC = 0.651) is a significantly better discriminator of FSHD status than any of the three DUX4 biomarkers (AUC < 0.557).

### PAX7 target gene repression is a superior biomarker to DUX4 target gene expression on single-cell RNA-Seq of FSHD myocytes

We obtained gene counts for the data described by van den Heuvel *et al*. (2019) (32) from the GEO database (33), accession GSE122873 (32). van den Heuvel *et al*. (32) performed single cell RNA-Seq on a total of 5133 primary cells differentiated *ex vivo* into unfused myocytes, derived from two FSHD1 and two FSHD2 patients; and 1914 myocytes from two control individuals.

We computed our PAX7 target gene repression biomarker and the three DUX4 target gene expression biomarkers (28) for each cell from the pooled FSHD cells versus the control cells. PAX7 target genes were significantly repressed in single cells from FSHD patients compared to controls (Wilcoxon *P* < 2.2 × 10^−16^; Fig. 3A), with all cells expressing some level of PAX7 target genes. Regarding DUX4, we found that the full Yao *et al*. (2014) (30) 114 DUX4 target gene expression signature, and our two un-patented DUX4 target gene expression signatures (28), were elevated in FSHD cells (Wilcoxon *P* < 0.005; Fig. 3B–D). However, this difference was largely driven by a small fraction of DUX4 target gene-positive FSHD cells, with the vast majority of both FSHD and control cells not expressing DUX4 target genes. This in line with the findings of van den Heuvel *et al*. (2019) (32) obtained using a 67 DUX4 target gene subset (DUX4-67 gene set) of the full Yao *et al*. (2014) (30) DUX4 target gene signature, where 0.4% (23/5133) of the FSHD cells had ≥5 DUX4 target genes, but none of the controls. Using the full Yao *et al*. (2014) (30) 114 gene DUX4 target biomarker, we found 19.7% of FSHD single cells and 7.8% of control cells had some detectable level of DUX4 target gene expression, again emphasizing the importance of using the full validated biomarkers.

Importantly, ROC curve analysis of our PAX7 target gene repression and the three DUX4 biomarkers on the single-cell RNA-Seq data from the van den Heuvel *et al*. (2019) (32) study demonstrated that PAX7 target gene repression was a powerful biomarker of FSHD myocytes, with an AUC of 0.651, compared to an AUC < 0.557 for the three DUX4 target gene biomarkers (Fig. 3E). Testing the comparative power of these four biomarkers over the same cells revealed that PAX7 target gene repression (AUC = 0.651) was significantly better at distinguishing FSHD cells from healthy cells (DeLong’s test of *P* < 2.2 × 10^−16^) than each of the three DUX4 target gene expression biomarkers (Fig. 3E).

### PAX7 target gene repression discriminates DUX4 target gene-negative FSHD myocytes from controls

**Figure 4:**
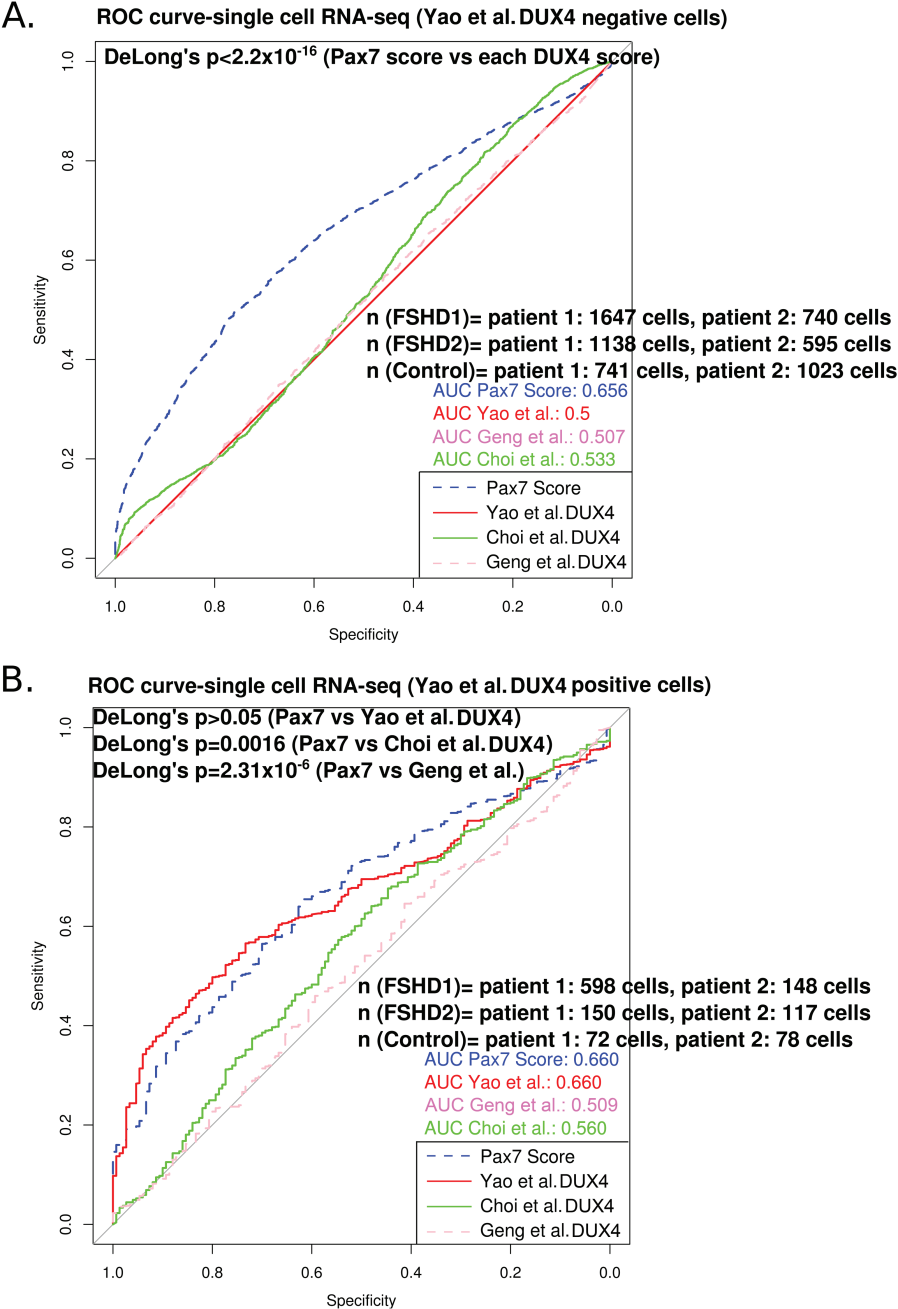
The PAX7 target gene repression biomarker can discriminate DUX4 target gene-negative FSHD myocytes from controls. (**A**) A ROC curve compares the discriminatory power of our PAX7 biomarker with the DUX4 target gene signatures of Yao *et al*. (2014) (30), Geng *et al*. (2012) (24) and Choi *et al*. (2016) (34), across just the single cells that showed no expression of the Yao *et al*. (2014) (30) 114 gene DUX4 signature, as sequenced from two FSHD1, two FSHD2 and two control individuals, published by van den Heuvel *et al*. (2019) (32). DeLong’s test *P-*value (*P* < 2.2 × 10^−16^) demonstrates that the PAX7 biomarker (AUC = 0.656) is a significantly better discriminator of FSHD status than each of the DUX4 biomarkers (AUC < 0.533). (**B**) A ROC curve compares the discriminatory power of our PAX7 biomarker with the DUX4 target gene signatures of Yao *et al*. (2014) (30), Geng *et al*. (2012) (24) and Choi *et al*. (2016) (34), across single cells that expressed the Yao *et al*. (2014) (30) DUX4 signature, as sequenced from two FSHD1, two FSHD2 and two control individuals, published by van den Heuvel *et al*. (2019) (32). DeLong’s test *P-*value (*P* = 0.99) demonstrates that the PAX7 biomarker (AUC = 0.660) is an equivalent discriminator of FSHD status to the Yao *et al*. (2014) (30) DUX4 signature (AUC = 0.660) on this pre-selected DUX4 target gene expressing population, but superior even to our other two DUX4 signatures (DeLong’s *P* < 0.0016).

Using the full Yao *et al*. (2014) (30) 114 gene DUX4 target signature, the large majority of both FSHD (80.3%) and control cells (92.2%) do not express DUX4 target genes. Thus, on these cells, DUX4 biomarkers provide no information for the classification of FSHD status. We considered just Yao *et al.* DUX4 target gene-negative cells and found that PAX7 target gene repression remained a significant biomarker of FSHD status (AUC = 0.656; Fig. 4A). On the 19.7% subset of Yao *et al*. (2014) (30) DUX4 target gene-positive FSHD cells, the PAX7 target gene repression signature was an equivalent biomarker to the Yao *et al*. (2014) (30) DUX4 target gene expression biomarker (PAX7 AUC = 0.660, DUX4 AUC = 0.660, DeLong’s *P* = 0.99; Fig. 4B). This implies that even on this biased subset where the discriminatory power of DUX4 target gene expression is maximized, it is still not a superior biomarker to PAX7 target gene repression. Indeed, the PAX7 target gene repression signature was significantly better than our DUX4 biomarkers derived from Geng *et al*. (2012) (24) and Choi *et al*. (2016) (34) in this pre-selected DUX4 target gene expressing population (DeLong’s *P* < 0.0016; Fig. 4B).

These results demonstrate that PAX7 target gene repression hallmarks FSHD myocytes regardless of their DUX4 target gene status, working effectively on both DUX4 target gene-positive or DUX4 target gene-negative cells. This biomarker may thus represent a more reliable discriminator than DUX4 target gene expression, especially when tissue availability is limited.

### A simple pipeline to evaluate established FSHD transcriptomic biomarkers

**Figure 5:**
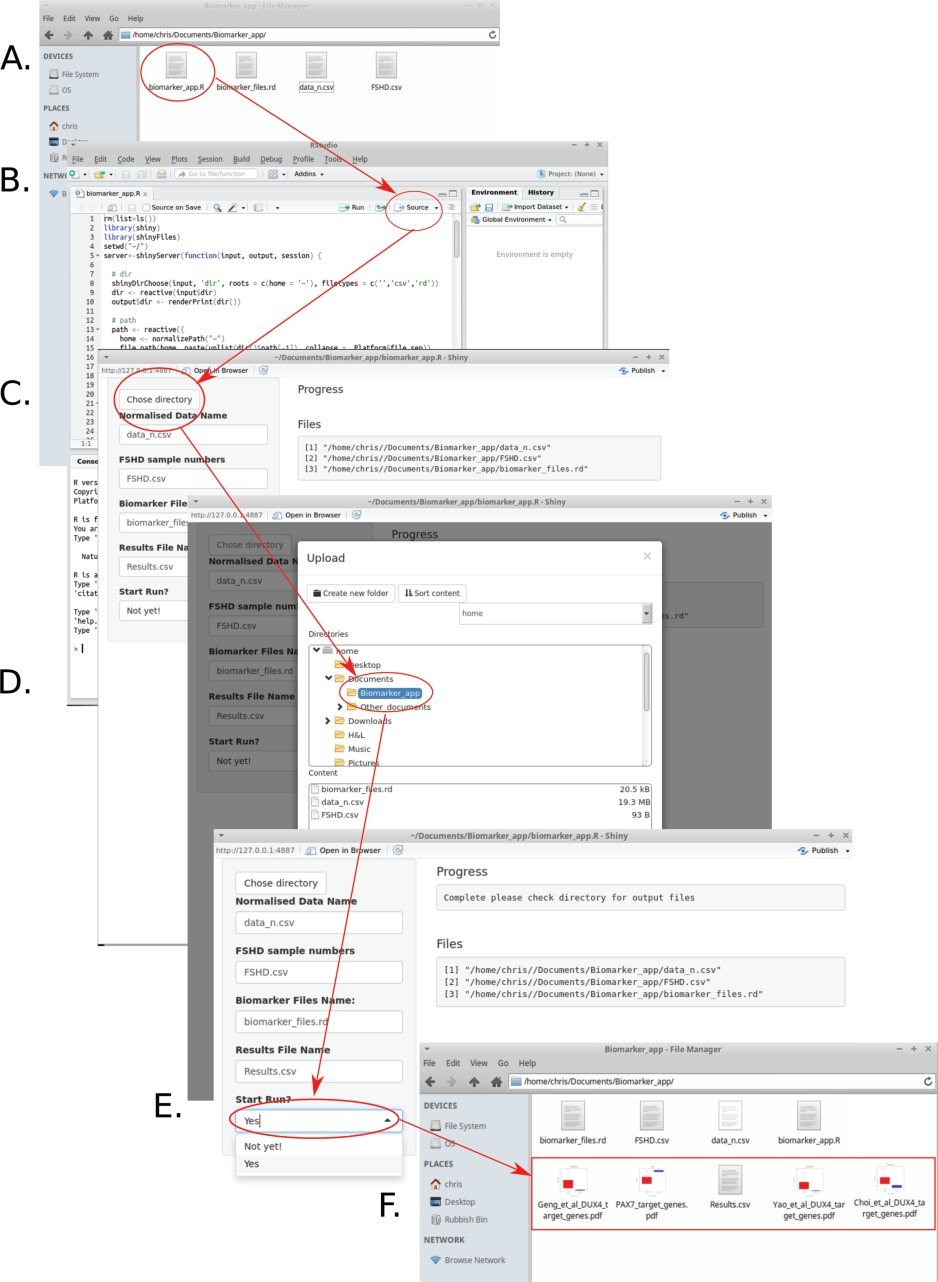
A simple pipeline to evaluate established FSHD transcriptomic biomarkers. Given a log-normalized data set describing Ensemble gene ID matched gene expression for FSHD and control samples alongside a list of which samples are from FSHD individuals, the PAX7 target gene repression biomarker and DUX4 target gene signatures of Yao *et al*. (2014) (30), Geng *et al*. (2012) (24) and Choi *et al*. (2016) (34) can be computed in six simple steps using our software (Supplementary Material, File S1). (**A**) Place the files describing the data and list of FSHD sample column numbers, in a directory alongside the files biomarker_files.Rd and biomarker_app.R obtainable as part of Supplementary Material, File S1 and open the file named biomarker_app.R. (**B**) Run the full script by clicking source if opening in R-studio (not necessary if opening in R), the GUI will open. (**C**) Select choose directory to select the directory containing the data file and FSHD sample numbers. (**D**) Once the directory is selected the program will display the file locations under the heading ‘Files’, confirming that the biomarker evaluation can commence. (**E**) Select ‘Yes’ from the ‘Start Run?’ dropdown menu to evaluate the biomarkers, once evaluated a message of completion will appear under the heading ‘Progress’. (**F**) Five files will be deposited in the selected directory: Results.csv (a table of the four biomarkers evaluated for each sample––can be opened in a spreadsheet program such as excel). Four box plots displaying the biomarker values for FSHD labelled samples versus the remaining samples in the data set (assumed controls). The *P*-value of a Wilcoxon test evaluating biomarker value differences between FSHD and control samples is shown beneath each plot.

Use of transcriptomic biomarkers in FSHD has the potential to aid disease staging, facilitate therapeutic development and support clinical trials. For such applications, however, biomarkers must be well validated and consistent. We previously evaluated our PAX7 target gene repression biomarker alongside three DUX4 target gene expression biomarkers across seven independent transcriptomic data sets of FSHD muscle biopsies and immortalized cell lines (28). Here we have evaluated these standardized PAX7 or DUX4 biomarkers on two further RNA-Seq-based transcriptomic data sets, particularly demonstrating the power of PAX7 target gene repression. To simplify use of these four established FSHD biomarkers that have now been evaluated over nine independent FSHD transcriptomic data sets (with PAX7 target gene repression the only biomarker achieving significance on 9/9 data sets), we have written a code for evaluating the PAX7 target gene repression biomarker and each of the three DUX4 target gene expression biomarkers (Supplementary Material, File S1). This was placed in a graphical user interface (GUI) wrapper using the shiny (36) and shinyFiles (37) packages in R, with the full program stored as a single file executable in R: ‘biomarker_app.R’, so can be run as an app. The app takes as input a normalized log-transformed table where rows are genes (annotated with Ensemble gene IDs) and columns are samples and outputs a file containing the PAX7 target repression biomarker values and the three separate DUX4 target gene expression biomarker (28) values for each sample (Fig. 5). We hope that this pipeline facilitates the use of consistent and validated biomarkers in FSHD transcriptomic studies.

## Discussion

We have previously demonstrated that PAX7 target gene repression hallmarks FSHD skeletal muscle (28). Our analysis here raises two important novel points for FSHD therapeutic development and biomarker utilization. Firstly, RNA-Seq data from MRI-guided muscle biopsies shows that PAX7 target gene repression correlates with histopathological markers of active disease in a manner independent of DUX4 target gene expression. FSHD muscle biopsy samples with high DUX4 target gene expression and high PAX7 target gene repression had more than double the histological evidence of active disease compared to samples with high DUX4 expression and low PAX7 target gene repression. This shows that DUX4 target gene expression is not the sole driver of active disease in FSHD and that PAX7 target gene repression may represent a viable therapeutic target. Currently, therapeutic development in FSHD largely focuses on inhibition of DUX4 expression with the aim of minimizing the pro-apoptotic effect of DUX4 target gene expression, and several molecules are in development with efficacy currently unclear (31,32,38–42). Our analysis suggests that while DUX4 target gene expression is associated with active disease, it does not fully account for pathology. Moreover, as we have also seen in our study, the overwhelming majority of cells isolated from FSHD patients express no detectible DUX4 or DUX4 target genes, raising the question of what the anti-DUX4 therapies are actually targeting. For example, even with DUX4 at barely detectible levels, FSHD myoblasts are highly sensitive to oxidative stress (20,43–45), and we have shown previously that PAX7 target gene repression is associated with up-regulation of HIF1α and an oversensitivity to oxidative stress (19,28). We have also shown *in vitro* efficacy of compounds that increase activity of mitochondrial biogenesis pathways in rescuing FSHD myotube hypotrophy (45), indicating that targeting oxidative stress sensitivity is of therapeutic relevance in FSHD (46). This indicates that targeting PAX7 target gene repression may be of therapeutic benefit in FSHD, in addition to reducing expression of DUX4 and its target genes.

Secondly, we demonstrate that PAX7 target gene repression can efficiently discriminate FSHD cells from controls, even when those cells do not express DUX4 target genes. The overwhelming majority of FSHD cells have no detectable levels of DUX4 target gene expression; with only 19.7% identified using the full Yao *et al*. (2014) DUX4 biomarker (30), while van den Heuvel *et al*. (2019) (32) reported only 0.4% of FSHD cells expressed ≥5 DUX4 target genes using a 67 gene subset of their full Yao *et al*. (2014) (30) DUX4 target gene signature. That PAX7 target gene repression can discriminate DUX4 target gene-negative FSHD cells from controls is important for at least two reasons. Anti-DUX4 therapy cannot be expected to target cells that do not express DUX4 or DUX4 target genes, but as we have shown, these cells still repress PAX7 targets and such repression is associated with a higher level of active disease. Thus, 80.3% of FSHD patient cells exhibit an FSHD molecular pathomechanism that anti-DUX4 therapy cannot be expected to ameliorate. This motivates the search for additional therapies. Secondly, muscle biopsies required for the evaluation of transcriptomic biomarkers are painful undertakings for patients with already damaged, inflamed muscle. Therefore, it is prudent that samples are as minimal as possible, particularly in clinical trials where repeated measurements may be required. For a biomarker to be of use in this circumstance, it must be robust on limited tissue samples; in the case of DUX4 target gene expression biomarkers there is a clear limit to how small a sample one can obtain before the signal is lost. In contrast, the PAX7 target gene repression biomarker can be readily measured on all FSHD cells isolated by van den Heuvel *et al*. (2019) (32) and is a significantly better classifier than biomarkers based on DUX4 target gene expression with a better scalability as biopsy size drops.

FSHD transcriptomic biomarkers will aid disease staging, facilitate therapeutic development and support clinical trials. We have now evaluated our PAX7 target gene repression biomarker alongside the full Yao *et al*. (2014) (30) and our two DUX4 target gene expression biomarkers across nine independent FSHD transcriptomic data sets here and previously (28). This has demonstrated the power, in particular, of PAX7 target gene repression in discriminating FSHD, being the only biomarker significant on 9/9 data sets. Although all four biomarkers are available as supplementary data to our previous publication (28), we were disappointed to see that subsequent FSHD transcriptomic studies [e.g. (31,32)] chose to employ only unvalidated subsets of the patented DUX4 target gene biomarker described by Yao *et al*. (2014) (30), and even then, used different subsets between publications. Analysis of our PAX7 biomarker was overlooked (31,32). Use of unvalidated and variant biomarkers without rigorous comparison makes it difficult to gain the consistency required for a biomarker to become a useful clinical tool. Here we evaluated all four validated biomarkers on these new transcriptomic studies and found PAX7 target gene repression to be the most robust in discriminating FSHD from control on single-cell RNA-Seq data, while all four biomarkers were equivalent on MRI-guided muscle biopsies. To facilitate investigators in evaluating these validated biomarkers, we have produced a GUI, code-free pipeline to obtain biomarker values from normalized gene expression data (Supplementary Material, File S1; Fig. 5).

In summary we have demonstrated that PAX7 target gene repression associates with active disease in FSHD independently of DUX4 target gene expression. At the single-cell level, our PAX7 target gene repression biomarker is a better discriminator of FSHD versus control cells, and can even discriminate DUX4 target gene-negative FSHD cells from controls. These findings have clear implications for therapeutic development and biomarker utilization.

## Materials and Methods

### Data set details and acquisition

Wang *et al*. (2019) (31) performed MRI-guided muscle biopsies from 6 quadriceps, 14 gastrocnemius, 13 tibialis anterior and 1 hamstring from 34 FSHD patients and from 9 control quadriceps, followed by histopathology-based scoring for the severity of the pathologic changes (haematoxylin and eosin, and trichrome, staining) and transcriptomic analysis using RNA-Seq (details in Supplemental Table S1 of Wang *et al*. (2019) (31)). Normalized gene level data from these 34 FSHD patients (12 female/22 male; mean age, 53.6 years; range, 20–75 years) and 9 controls (6 female/3 male; mean age, 35 years; range, 19–56 years) described by Wang *et al*. (2019) (31) were downloaded from the GEO database (33), accession GSE115650 (31). Data were log transformed and quantile normalized to ensure compatibility with our previous evaluations of FSHD biomarkers (28).

Normalized gene level data for the single-cell RNA-Seq corresponding to *ex vivo* 48–72 h differentiated, unfused (via calcium chelation with ethylene glycol tetraacetic acid), myocyte cultures from two FSHD1 (one female/one male), two FSHD2 (two female) and two control (two female) primary myoblast lines described by van den Heuvel *et al*. (2019) (32) were downloaded from the GEO database (33), accession GSE122873 (32). Data were log transformed and quantile normalized to ensure compatibility with our previous evaluations of FSHD biomarkers (28).

### Biomarker evaluation

Our PAX7 target gene repression biomarker is derived from 311 up-regulated PAX7 target genes and 290 down-regulated PAX7 target genes in mouse (28). For DUX4, the full 114 DUX4 target gene signature of Yao *et al*. (2014) (30) and our 165 DUX4 target gene signature derived from Geng *et al*. (2012) (24) and 212 DUX4 target gene signature from Choi *et al*. (2016) (34) were evaluated as previously described (28). Briefly, the PAX7 target gene repression score for each sample was computed as the *t-*score from a test comparing the up-regulated to down-regulated PAX7 target genes within each sample. Each DUX4 target gene expression score is computed for each sample as the mean expression of the genes found to be up-regulated by the studies of Yao *et al*. (2014) (30), Geng *et al*. (2012) (24) or Choi *et al*. (2016) (34). Score differences between FSHD and controls samples were evaluated within each study via a Wilcoxon U-test. ROC curve analysis and DeLong’s testing were performed using the pROC package in R (47). For FSHD muscle biopsy samples described by Wang *et al*. (2019) (31) univariate linear models for histopathological measures of active disease and inflammation were fit for each of the biomarkers in turn, as well as the average of the three DUX4 target gene expression biomarkers for each sample using the base package in R. Multivariate linear models for active disease and inflammation were fit using both PAX7 target gene repression and average DUX4 target gene expression as separate covariates to demonstrate independent associations also using the base package in R.

### Biomarker evaluation software

The code for evaluating the PAX7 target gene repression biomarker and each of the three DUX4 target gene expression biomarkers (Supplementary Material, File S1) was placed in a GUI wrapper using the shiny (36) and shinyFiles (37) packages in R, with the full program stored as a single file executable in R: ‘biomarker_app.R’. The software takes as input two files: one describing a table of log-normalized data where rows contain Ensemble gene IDs and columns are samples and a second file describing a single column containing the column numbers of samples in the data file that are from FSHD individuals (with the remainder assumed controls). The software also has a dependency file ‘biomarker_files.Rd’, which is an RData file containing the genes required for the evaluation of each of the biomarkers. The program outputs five files: Results.csv (a table of the four biomarkers evaluated for each sample in the data set) and four box plots displaying the biomarker values for FSHD labelled samples versus the remaining samples in the data set (assumed controls). The *P*-value of a Wilcoxon test evaluating biomarker value differences between FSHD and control samples is shown beneath each plot. A .zip file containing ‘biomarker_app.R’, ‘biomarker_files.Rd’ and the data of Wang *et al*. (2019) (31) evaluated in this paper, provided as an example, alongside a README file are provided as Supplementary Material, File S1.

*Conflict of Interest statement.* None declared.

## Supplementary Material

Supplementary_Data_ddz043
