# Supplementary material for: PAX7 target gene repression is a superior FSHD biomarker than DUX4 target gene activation, associating with pathological severity and identifying FSHD at the single-cell level
Source: Hum Mol Genet. 2019 Mar 13;28(13):2224–36. doi: 10.1093/hmg/ddz043 (PMC6586142; doi:10.1093/hmg/ddz043)
Supplement: Supplementary_Data_ddz043 [file supplementary_data_ddz043.zip › README.docx]

README for Biomarker_app

1. Download and install R (free version) appropriate to your operating system from the CRAN repository https://cran.r-project.org/

2. Optional: install R-studio (free version) from https://www.rstudio.com/products/rstudio/download/

3. Open R/R-studio and install the R packages, 'devtools', 'shiny' and 'shinyFiles' version 0.6.2 unsing the following lines (copy and paste directly into the terminal)

install.packages('devtools')

install.packages('shiny')

require(devtools)

install_version("shinyFiles", version = "0.6.2", repos = "http://cran.us.r-project.org")

4. Obtain the following files:

data_n.csv (csv table of log-normalised gene expression data with first column Ensemble gene IDs and remaining columns are samples)

FSHD.csv (a column containing numbers corresponding to which samples are FSHD in data_n.csv)

Both csv files can be compiled in a spreadsheet program such as excel, via Save As, file type=CSV

biomarker_files.rd (an RData file containing the FSHD biomarker genes obtained as supplementary data from Banerji et al., 2019)

biomarker_app.R (an R script containing the biomarker evaluated obtained as supplementary data from Banerji et al., 2019)

5. Open the file Biomarker_app.R in R/R-studio and run the full script (double click the icon and click source if using R-studio)

6. The GUI window will open. Click on choose directory to select the folder where the files data_n.csv, FSHD.csv and biomarker_files.Rd are located - if the program can locate these files they will be shown under 'Files' on the GUI

7. If the names of the input files are different to those given above the user can input these names to be located by the program, the user can also select the name of the results file containing the biomarker values for each sample

8. Switch start run from 'Not yet!' to 'Yes and the biomarkers will be evaluated, once evaluated a message of completion will appear under 'Progress'

9. 5 files will be deposited in the selected directory: Results.csv (a table of the 4 biomarkers evaluated for each sample - can be opened in a spreadsheet program such as excel). Four boxplots displaying the biomarker values for FSHD labelled samples vs the remaining samples in the data set (assumed controls). The *p*-value of a Wilcoxon Test evaluating biomarker value differences between FSHD and control samples is shown beneath each plot.
